# Correction: Patient-specific computational simulation of coronary artery bypass grafting

**DOI:** 10.1371/journal.pone.0318664

**Published:** 2025-01-29

**Authors:** The PLOS ONE Staff

The authors are listed out of order. Please view the correct author order, affiliations, and citation here:

Wei Wu ^1,2^, Mohammadali Sharzehee^1^, Anastasios Nikolaos Panagopoulos^1^, Charu Hasini Vasa^1,2^, Shijia Zhao^1,2^, Saurabhi Samant^1^, Usama M. Oguz^1,2^, Behram Khan^1^, Abdallah Naser^1^, Khaled M. Harmouch^1^, Ghassan S. Kassab^3^, Aleem Siddique^4^, Yiannis S. Chatzizisis^1,2^

**1** Cardiovascular Biology and Biomechanics Laboratory, Cardiovascular Division, University of Nebraska Medical Center, Omaha, Nebraska, United States of America, **2** Division of Cardiovascular Medicine, Miller School of Medicine, University of Miami, Miami, Florida, United States of America, **3** California Medical Innovation Institute, San Diego, California, United States of America**, 4** Division of Cardiothoracic Surgery, University of Nebraska Medical Center, Omaha, Nebraska, United States of America.

Wu W, Sharzehee M, Panagopoulos AN, Vasa CH, Zhao S, Samant S, et al. (2023) Patient-specific computational simulation of coronary artery bypass grafting. PLOS ONE 18(3): e0281423. https://doi.org/10.1371/journal.pone.0281423.

The publisher apologizes for the errors.

## References

[1] Wu W, Panagopoulos AN, Vasa CH, Sharzehee M, Zhao S, Samant S, et al. (2023) Patient-specific computational simulation of coronary artery bypass grafting. PLOS ONE 18(3): e0281423. 10.1371/journal.pone.0281423 36867601 PMC9983828

