# Supplementary material for: Patient-specific computational simulation of coronary artery bypass grafting
Source: PLoS One. 2023 Mar 3;18(3):e0281423. doi: 10.1371/journal.pone.0281423 (PMC9983828; doi:10.1371/journal.pone.0281423)
Supplement: S1 Table — (DOCX) [file pone.0281423.s001.docx]

**S1 Table.** Clinical, angiographic, and graft characteristics of the study patients

|  | **Aim 1** | | **Aim 2** | |
| --- | --- | --- | --- | --- |
| **Patient number** | **1** | **2** | **3** | **4** |
| **Clinical characteristics** | | |  | |
| Age (years) | 68 | 85 | 82 | 76 |
| Sex | Male | Male | Male | Female |
| Diabetes | No | Yes | Yes | Yes |
| Hyperlipidemia | Yes | Yes | Yes | Yes |
| Hypertension | Yes | No | Yes | Yes |
| Chronic kidney disease | No | No | No | Yes |
| Chronic obstructive pulmonary disease | No | No | No | Yes |
| Left ventricular ejection fraction | 55% | 40% | 45% | 50-55% |
| **Angiographic characteristics** |  | |  | |
| Baseline anatomy | LAD: occluded  (distal to D1)  D: 80% stenosis  LCX: 80% stenosis  RCA: 80% stenosis | LAD: 70% stenosis  D: 80% stenosis  OM: occluded  RCA: occluded | LAD: 60% stenosis  LCX: 80% stenosis D: 80% stenosis  PDA: 80% stenosis | LAD: 60% stenosis  RCA: 70% stenosis |
| **Graft characteristics** |  | |  | |
| Number of grafts | 5 | 4 | 4 | 1 |
| Type of grafts | - LIMA to LAD - SVG to OM - RIMA to PDA   (occluded)   - SVG (sequential) to OM1/PLV   (occluded) | - LIMA to LAD - SVG (Ysequential) to OM and PLV   (occluded)   - SVG to PDA | - LIMA to LAD - SVG to PDA - SVG (sequential) to LCX and D   (occluded) | - LIMA to LAD |

LAD: left anterior descending, D: diagonal, LCX: left circumflex, RCA: right coronary artery, LIMA: left internal mammary artery, SVG: saphenous vein graft, OM: obtuse marginal, RIMA: right internal mammary artery, PDA: posterior descending artery, PLV: posterior left ventricular
