# Supplementary material for: Patient-specific computational simulation of coronary artery bypass grafting
Source: PLoS One. 2023 Mar 3;18(3):e0281423. doi: 10.1371/journal.pone.0281423 (PMC9983828; doi:10.1371/journal.pone.0281423)
Supplement: S2 Table — (DOCX) [file pone.0281423.s002.docx]

| **S2 Table.** Total boundary parameters used in the patients | | | | | | | |
| --- | --- | --- | --- | --- | --- | --- | --- |
| Parameter values of non-coronary branches for Patient 1 | | | | | | | |
| Non-coronary branches | | R_p_ (dynes s/cm^5^) | | C (cm^5^/dynes) | | R_d_ (dynes s/cm^5^) | |
| RSCA  RCCA  LCCA  LSCA  Aorta outlet | | 2,295  2,801  3,107  2,371  189 | | 1.28E-04  1.05E-04  9.43E-05  1.24E-04  1.55E-03 | | 23,208  28,325  31,418  23,973  1,911 | |
| Parameter values of coronary branches for Patient 1 | | | | | | | |
| Coronary branches | R_a_  (dynes s/cm^5^) | | C_a_  (cm^5^/dynes) | R_a-micro_  (dynes s/cm^5^) | C_im_  (cm^5^/dynes) | | R_v_+R_v-micro_ (dynes s/cm^5^) |
| RCA  RCA1  RCA2  LAD  LAD1  LCX  LCX1 | 219,976  84,332  112,260  257,099  158,703  26,145  160,005 | | 4.96e-07  1.288e-06  9.67e-07  2.81e-07  4.56e-07  2.76e-06  4.52e-07 | 357,462  137,039  18,2423  41,7786  25,7893  42,486  260,008 | 3.99e-06  1.04e-05  7.83e-06  2.27e-06  3.69e-06  2.24e-05  3.66e-06 | | 109,988  42,166  56,130  128,549  79,351  13,072  80,002 |
| Parameter values of non-coronary branches for Patient 2 | | | | | | | |
| Non-coronary branches | | R_p_ (dynes s/cm^5^) | | C (cm^5^/dynes) | | R_d_ (dynes s/cm^5^) | |
| RSCA  RCCA  LCCA  LSCA  Aorta outlet | | 3,147  3,146  3,277  3,452  178 | | 9.31E-05  9.31E-05  8.94E-05  8.48E-05  1.64E-03 | | 31,822  31,814  33,138  34,921  1,806 | |
| Parameter values of coronary branches for Patient 2 | | | | | | | |
| Coronary branches | R_a_  (dynes s/cm^5^) | | C_a_  (cm^5^/dynes) | R_a-micro_  (dynes s/cm^5^) | C_im_  (cm^5^/dynes) | | R_v_+R_v-micro_ (dynes s/cm^5^) |
| LAD  LAD1  LAD2  LCX  LCX1 | 88,723  49,533  58,687  107,631  77,417 | | 5.57e-07  9.99e-07  8.43e-07  4.59e-07  6.39e-07 | 144,176  80,492  95,367  174,900  125,803 | 4.51e-06  8.08e-06  6.82e06  3.72e-06  5.17e-06 | | 44,361  24,766  29,343  53,815  38,708 |
| Parameter values of non-coronary branches for Patient 3 | | | | | | | |
| Non-coronary branches | | R_p_ (dynes s/cm^5^) | | C (cm^5^/dynes) | | R_d_ (dynes s/cm^5^) | |
| RSCA  RCCA  LCCA  LSCA  Aorta outlet | | 2,134  2,439  1,760  2,261  202 | | 1.37E-04  1.20E-04  1.66E-04  1.30E-04  1.45E-03 | | 21581  24662  17803  22865  2047 | |
| Parameter values of coronary branches for Patient 3 | | | | | | | |
| Coronary branches | R_a_  (dynes s/cm^5^) | | C_a_  (cm^5^/dynes) | R_a-micro_  (dynes s/cm^5^) | C_im_  (cm^5^/dynes) | | R_v_+R_v-micro_ (dynes s/cm^5^) |
| RCA  RCA1  RCA2  RCA3  LAD  LAD1  LAD2  LAD3  LAD4  LCX  LCX1 | 200,535  176,591  87,531  665,966  71,913  200,023  102,108  180,818  308,266  114,451  97,901 | | 5.81e-07  6.60e-07  1.33e-06  1.75e-07  9.75e-07  3.50e07  6.87e-07  3.88e-07  2.27e-07  6.13e-07  7.16e-07 | 325,869  286,960  142,239  108,2190  116,860  325,038  165,925  293,829  500,933  185,984  159,091 | 4.70e-06  5.34e-06  1.07e-05  1.41e-06  7.89e-06  2.83e-06  5.56e-06  3.14e-06  1.84e-06  4.96e-06  5.80e-06 | | 100,267  88,295  43,765  332,983  35,957  100,012  51,053  90,408  154,133  57,225  48,950 |
| Parameter values of non-coronary branches for Patient 4 | | | | | | | |
| Non-coronary branches | | R_p_ (dynes s/cm^5^) | | C (cm^5^/dynes) | | R_d_ (dynes s/cm^5^) | |
| RSCA  RCCA  LCCA  LSCA  Aorta outlet | | 4,266  1,598  3,080  1,995  194 | | 6.87E-05  1.83E-04  9.51E-05  1.47E-04  1.51E-03 | | 43,138  16,163  31,144  20,175  1,966 | |
| Parameter values of coronary branches for Patient 4 | | | | | | | |
| Coronary branches | R_a_  (dynes s/cm^5^) | | C_a_  (cm^5^/dynes) | R_a-micro_  (dynes s/cm^5^) | C_im_  (cm^5^/dynes) | | R_v_+R_v-micro_ (dynes s/cm^5^) |
| RCA  RCA1  LAD  LAD1  LAD2  LCX | 82,316  101,541  49,228  84,976  141,835  52,989 | | 1.51e-06  1.23e-06  1.38e-06  8.03-07  4.81e-07  1.28e-06 | 133,764  165,004  79,997  138086  230,481  86,107 | 1.22e-05  9.96e-06  1.12e-05  6.50e-06  3.89e-06  1.04e-05 | | 41,158  50,770  24,614  42,488  70,917  26,494 |
| Right Subclavian Artery, RCCA: Right Common Carotid Artery, LCCA: Left Common Carotid Artery, LSCA: Left Subclavian Artery, RCA: Right Coronary Artery, LAD: Left Anterior Descending Artery, LCX: Left Circumflex Artery, Rp: Resistance of proximal vessels, Ra: Coronary arterial resistance, Rd: Resistance of distal vessels, Ra-micro: Coronary arterial microcirculation resistance, Rv: Coronary venous resistance, Rv-micro: Coronary venous microcirculation resistance, C: Capacitance of proximal vessels, Ca: Coronary arterial compliance, Cim: Intramyocardial compliance | | | | | | | |

RSCA:
