# Supplementary material for: Patient-specific computational simulation of coronary artery bypass grafting
Source: PLoS One. 2023 Mar 3;18(3):e0281423. doi: 10.1371/journal.pone.0281423 (PMC9983828; doi:10.1371/journal.pone.0281423)
Supplement: S3 Table — (DOCX) [file pone.0281423.s003.docx]

**S3 Table.** Solver parameters used in the CFD simulation.

| **Parameter** | **Value** |
| --- | --- |
| Time Step Parameters | |
| Number of Timesteps | 12000 |
| Time Step Size | 0.0005 |
| Output Control | |
| Number of Timesteps between Restarts | 100 |
| Output Surface Stress | True |
| Force Calculation Method | Velocity Based |
| Print Average Solution | True |
| Print Error Indicators | False |
| Step Construction | |
| Step Construction | 5 |
| ===Advanced Parameters=== | |
| Pressure Coupling | Implicit |
| Backflow Stabilization Coefficient | 0.2 |
| Non-linear Iteration Control | |
| Residual Control | True |
| Residual Criteria | 0.01 |
| Minimum Required Iterations | 3 |
| Linear Solver | |
| svLS Type | NS |
| Number of Krylov Vectors per Left-hand-side Formation | 100 |
| Number of Solves per Left-hand-side Formation | 1 |
| Tolerance on Momentum Equations | 0.05 |
| Tolerance on Continuity Equations | 0.4 |
| Tolerance on svLS Solver | 0.4 |
| Maximum Number of Iterations for svLS NS Solver | 1 |
| Maximum Number of Iterations for svLS Momentum Loop | 2 |
| Maximum Number of Iterations for svLS Continuity Loop | 400 |
| Discretization Control | |
| Time Integration Rule | Second Order |
| Time Integration Rho Infinity | 0.5 |
| Flow Advection Form | Convective |
| Quadrature Rule on Interior | 2 |
| Quatrature Rule on Boundary | 3 |
