# Supplementary material for: Patient-specific computational simulation of coronary artery bypass grafting
Source: PLoS One. 2023 Mar 3;18(3):e0281423. doi: 10.1371/journal.pone.0281423 (PMC9983828; doi:10.1371/journal.pone.0281423)
Supplement: S4 Table — (DOCX) [file pone.0281423.s004.docx]

**S4 Table.** Hemodynamic parameter definitions.

| Hemodynamic Parameter | Definition |
| --- | --- |
| Time-averaged WSS | $\frac{1}{T}\int_{0}^{T} \left\vert\tau_{w} \right\vert dt$ |
| Oscillatory shear index | $0.5\left[ 1-\left\vert\int_{0}^{T} \tau_{w}dt \right\vert/\int_{0}^{T} \left\vert\tau_{w} \right\vert dt \right]$ |
| Computationally Resting P_d_/P_a_ | 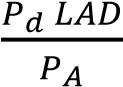 at resting condition |
| Computational FFR | 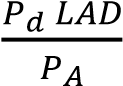at hyperemic condition |

T: duration of one cardiac cycle, $\tau_{w}$: wall shear stress (WSS), P_d_: mean distal pressure at the LAD distal and proximal to the computationally created stenosis, P_A:_ mean aortic pressure, FFR: fractional flow reserve
