# Supplementary material for: Patient-specific computational simulation of coronary artery bypass grafting
Source: PLoS One. 2023 Mar 3;18(3):e0281423. doi: 10.1371/journal.pone.0281423 (PMC9983828; doi:10.1371/journal.pone.0281423)
Supplement: S5 Table — (DOCX) [file pone.0281423.s005.docx]

**S5 Table.** Computational FFR measurement proximal and distal to the computationally created LAD stenosis pre- and post-CABG.

|  | **Computational FFR Proximal to the**  **LAD Stenosis** | | **Computational FFR Distal to the LAD Stenosis** | |
| --- | --- | --- | --- | --- |
|  | Pre-CABG | Post-CABG | Pre-CABG | Post-CABG |
| **Patient 3** |  |  |  |  |
| Mild Stenosis | 0.92 | 0.95 | 0.82 | 0.87 |
| Moderate Stenosis | 0.94 | 0.96 | **0.77** | 0.86 |
| Severe Stenosis | 0.95 | 0.97 | **0.62** | 0.82 |
| Critical Stenosis | 0.97 | 0.98 | **0.19** | **0.74** |
|  |  |  |  |  |
| **Patient 4** |  |  |  |  |
| Mild Stenosis | 0.96 | 0.97 | 0.88 | 0.91 |
| Moderate Stenosis | 0.96 | 0.98 | 0.83 | 0.90 |
| Severe Stenosis | 0.97 | 0.98 | **0.64** | 0.87 |
| Critical Stenosis | 0.98 | 0.98 | **0.14** | 0.81 |


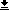


FFR: fractional flow reserve, LAD: left anterior descending, CABG: coronary artery bypass grafting
