# Supplementary material for: Patient-specific computational simulation of coronary artery bypass grafting
Source: PLoS One. 2023 Mar 3;18(3):e0281423. doi: 10.1371/journal.pone.0281423 (PMC9983828; doi:10.1371/journal.pone.0281423)
Supplement: Attachment — Submitted filename: Response to Reviewers.docx [file pone.0281423.s006.docx]

**Editor Comments:**

**The manuscript has several weaknesses and needs an extensive and careful revision.**

**I agree with the comments made by the reviewers and recommend the authors to take into account those comments and submit an improved version of the manuscript.**

In the revised version of the manuscript, we provided detailed clarifications and revisions, where we highlight the technical innovations of our work and the important clinical perspectives.

**Reviewer #1:**

**The paper deals with a CFD study for coronary by-passes. In particular, the authors compute the Fractional Flow Reserve (FFR) for two patients and compare it with angiography measures with good results. The paper is of interest for the journal, however some issues should be better discussed by the authors in the text before publication:**

We would like to thank the Reviewer for the critique.

**Comment 1: Introduction. The literature about computational studies for coronary by-passes should be improved, I suggest for example to include:**

**- Nordgaard H, Swillens A, Nordhaug D, Kirkeby-Garstad I, Van Loo D, Vitale N, Segers P, Haaverstad R, Lovstakken L,** **Impact of competitive flow on wall shear stress in coronary surgery: computational fluid dynamics of a LIMA-LAD model. Cardiovasc Res, 2010**

**- Guerciotti B., Vergara C., Ippolito S., Quarteroni A., Antona C., Scrofani R.,** **Computational study of the risk of restenosis in coronary bypasses. Biomechanics and Modeling in Mechanobiology, 2017**

**Response:** As suggested, we added the following references to the text in the Introduction (page 3).

**Comment 2: Related to the previous point: collocate the paper with respect to the previous works done on the topic: which is the novelty, what is common to other works? In general the first part of Introduction is too technical and long, I would rather focus on contestualization of the paper.**

**Response:** As suggested, we significantly shortened the 2^nd^ paragraph of the Introduction (pages 2-3).

Also in the Introduction, we highlight the novelty of our work in the context of the current state-of-the-art (page 3):

*“A study investigated the competitive flow using computational fluid dynamics in animal models [18]. Another study performed a parametric computational analysis of a relationship between the degree of coronary artery stenosis and the risk of graft failure [21]. Other studies used a more sophisticated approach of fluid-structure interaction to explore the association of blood flow and stress in bypass grafts and native arteries [22, 23]. There have also been some recent attempts to use patient-specific CFD to study the flow physiology in the stenotic native coronary artery and virtually implanted grafts [24].”*

**Comment 3: a) To complete the state of the art about the topic, authors should also mention the works that focused on FSI for coronary by-passes, e.g. I suggest to include:**

**- Kabinejadian F, Ghista DN (2012)** **Compliant model of a coupled sequential coronary arterial bypass graft: effects of vessel wall elasticity and non-newtonian rheology on blood flow regime and hemodynamic parameters distribution. Med Eng Phys, 2012**

**-Guerciotti B., Vergara C., Ippolito S., Quarteroni A., Antona C., Scrofani R.,** **A computational fluid-structure interaction analysis of coronary Y-grafts. Medical Engineering & Physics, 2017**

**b) Moreover, the authors should discuss in the Limitations section the assumption of rigid walls**

**Response:** We cited the suggested references in the Introduction (page 3):

*“A study investigated the competitive flow using computational fluid dynamics in animal models [18]. Another study performed a parametric computational analysis of a relationship between the degree of coronary artery stenosis and the risk of graft failure [21].* ***Other studies used a more sophisticated approach of fluid-structure interaction to explore the association of blood flow and stress in bypass grafts and native arteries [22, 23].*** *There have also been some recent attempts to use patient-specific CFD to study the flow physiology in the stenotic native coronary artery and virtually implanted grafts [24].”*

Also, we modified the assumption of rigid walls in the Limitations (section 4.4, page 15):

*“Sixth, there were a series of limitations related to our computational methodology: (i) The values of the aortic flow rates and ventricular pressures were derived from published data corresponding to healthy individuals and not to individual study patients [27, 29]. (ii) To reduce the complexity of computational calculations, we used an open-loop model with the same lumped parameters in the pre- and post-CABG simulations [28], (iii)* ***In simulations, the vascular wall was considered rigid; however the capacitance was distributed proportionally to the outlets to capture the global effects of flexible walls [29].****”*

**Comment 4: "Aim 1: To describe and test a new patient-specific computational framework"**

**In which sense "new"? Here and later in the Methods the authors should be more precise on this point**

**Response:** We thank the Reviewer for giving us the opportunity to clarify this important point. What is new in our work is that we use existing and well-validated technologies e.g., CCTA, CFD, FFR to create a framework that can guide clinical-decision making of bypass surgery. Essentially, we repurpose existing tools towards a new clinical application. These concepts the novelty of our work is clarified in the Introduction (page 3):

*“This study had two Aims:* ***Aim 1****: To describe and test a new patient-specific computational framework that combines CCTA with CFD to calculate the local hemodynamics in native coronary arteries and bypass grafts, and* ***Aim 2****: To use the tested computational framework of Aim 1, to perform computational hemodynamic investigations on the:* ***(i)*** *Effect of bypass grafting on the local hemodynamics in the native artery, and* ***(ii)*** *Hemodynamic interdependence (competitive flow) between the graft and native coronary artery.”*

Also, we highlight this point in the Discussion (page 12):

*“The novelty in our work is that we repurposed existing computational modalities and technologies (CCTA, CFD, FFR) to build a new framework and clinical application that* *could serve as a clinically impactful tool for effective surgical planning based on non-invasive hemodynamic assessment of coronary artery disease.”*

**Comment 5: Methods: "... computationally created focal lumen stenoses with four degrees of severity (mild, moderate,severe, critical)"**

**Please cite previous works where different degrees of stenosis were virtually created, e.g.**

**- Guerciotti B., Vergara C., Ippolito S., Quarteroni A., Antona C., Scrofani R., Computational study of the risk of restenosis in coronary bypasses. Biomechanics and Modeling in Mechanobiology, 2017**

**Response:** We cited the suggested references in the Methods (section 2.3.1, page 7):

***“****We 3D reconstructed these anatomies, as described in 2.2.1, and then computationally created focal lumen stenosis in the reconstructed LAD with four degrees of diameter reduction i.e., mild (29%), moderate (50%), severe (68%), and critical (82%) [20].****”***

**Comment 6: Page 8: "The boundary condition values were tuned until ..."**

**In which sense "bc values"? What is tuned? The values of parameters (R,C,...)? Please provide more details on the calibration phase and report the final parameters values used in the simulations.**

**Response:** We would like to thank the Reviewer for raising this point related to the calibration of boundary conditions. We provide details on this in the Methods (section 2.2.2, page 6):

*“The resistance (Rp, Rd…) and capacitance (C…) of each patient’s outlet were iteratively and manually adjusted to match the normal aortic root blood pressure of 120/80 mmHg. These boundary condition values were tuned until the difference to the target pressure of the aorta was less than 5 mmHg. The resistance and capacitance parameters for each patient are listed in* ***Supplemental Table 2****.”*

Also, we summarized the individual boundary parameters for each patient in **Supplemental Table 2**.

| **Supplemental Table 2.** Total boundary parameters used in the patients | | | | | | | |
| --- | --- | --- | --- | --- | --- | --- | --- |
| Parameter values of non-coronary branches for Patient 1 | | | | | | | |
| Non-coronary branches | | R_p_ (dynes s/cm^5^) | | C (cm^5^/dynes) | | R_d_ (dynes s/cm^5^) | |
| RSCA  RCCA  LCCA  LSCA  Aorta outlet | | 2,295  2,801  3,107  2,371  189 | | 1.28E-04  1.05E-04  9.43E-05  1.24E-04  1.55E-03 | | 23,208  28,325  31,418  23,973  1,911 | |
| Parameter values of coronary branches for Patient 1 | | | | | | | |
| Coronary branches | R_a_  (dynes s/cm^5^) | | C_a_  (cm^5^/dynes) | R_a-micro_  (dynes s/cm^5^) | C_im_  (cm^5^/dynes) | | R_v_+R_v-micro_ (dynes s/cm^5^) |
| RCA  RCA1  RCA2  LAD  LAD1  LCX  LCX1 | 219,976  84,332  112,260  257,099  158,703  26,145  160,005 | | 4.96e-07  1.288e-06  9.67e-07  2.81e-07  4.56e-07  2.76e-06  4.52e-07 | 357,462  137,039  18,2423  41,7786  25,7893  42,486  260,008 | 3.99e-06  1.04e-05  7.83e-06  2.27e-06  3.69e-06  2.24e-05  3.66e-06 | | 109,988  42,166  56,130  128,549  79,351  13,072  80,002 |
| Parameter values of non-coronary branches for Patient 2 | | | | | | | |
| Non-coronary branches | | R_p_ (dynes s/cm^5^) | | C (cm^5^/dynes) | | R_d_ (dynes s/cm^5^) | |
| RSCA  RCCA  LCCA  LSCA  Aorta outlet | | 3,147  3,146  3,277  3,452  178 | | 9.31E-05  9.31E-05  8.94E-05  8.48E-05  1.64E-03 | | 31,822  31,814  33,138  34,921  1,806 | |
| Parameter values of coronary branches for Patient 2 | | | | | | | |
| Coronary branches | R_a_  (dynes s/cm^5^) | | C_a_  (cm^5^/dynes) | R_a-micro_  (dynes s/cm^5^) | C_im_  (cm^5^/dynes) | | R_v_+R_v-micro_ (dynes s/cm^5^) |
| LAD  LAD1  LAD2  LCX  LCX1 | 88,723  49,533  58,687  107,631  77,417 | | 5.57e-07  9.99e-07  8.43e-07  4.59e-07  6.39e-07 | 144,176  80,492  95,367  174,900  125,803 | 4.51e-06  8.08e-06  6.82e06  3.72e-06  5.17e-06 | | 44,361  24,766  29,343  53,815  38,708 |
| Parameter values of non-coronary branches for Patient 3 | | | | | | | |
| Non-coronary branches | | R_p_ (dynes s/cm^5^) | | C (cm^5^/dynes) | | R_d_ (dynes s/cm^5^) | |
| RSCA  RCCA  LCCA  LSCA  Aorta outlet | | 2,134  2,439  1,760  2,261  202 | | 1.37E-04  1.20E-04  1.66E-04  1.30E-04  1.45E-03 | | 21581  24662  17803  22865  2047 | |
| Parameter values of coronary branches for Patient 3 | | | | | | | |
| Coronary branches | R_a_  (dynes s/cm^5^) | | C_a_  (cm^5^/dynes) | R_a-micro_  (dynes s/cm^5^) | C_im_  (cm^5^/dynes) | | R_v_+R_v-micro_ (dynes s/cm^5^) |
| RCA  RCA1  RCA2  RCA3  LAD  LAD1  LAD2  LAD3  LAD4  LCX  LCX1 | 200,535  176,591  87,531  665,966  71,913  200,023  102,108  180,818  308,266  114,451  97,901 | | 5.81e-07  6.60e-07  1.33e-06  1.75e-07  9.75e-07  3.50e07  6.87e-07  3.88e-07  2.27e-07  6.13e-07  7.16e-07 | 325,869  286,960  142,239  108,2190  116,860  325,038  165,925  293,829  500,933  185,984  159,091 | 4.70e-06  5.34e-06  1.07e-05  1.41e-06  7.89e-06  2.83e-06  5.56e-06  3.14e-06  1.84e-06  4.96e-06  5.80e-06 | | 100,267  88,295  43,765  332,983  35,957  100,012  51,053  90,408  154,133  57,225  48,950 |
| Parameter values of non-coronary branches for Patient 4 | | | | | | | |
| Non-coronary branches | | R_p_ (dynes s/cm^5^) | | C (cm^5^/dynes) | | R_d_ (dynes s/cm^5^) | |
| RSCA  RCCA  LCCA  LSCA  Aorta outlet | | 4,266  1,598  3,080  1,995  194 | | 6.87E-05  1.83E-04  9.51E-05  1.47E-04  1.51E-03 | | 43,138  16,163  31,144  20,175  1,966 | |
| Parameter values of coronary branches for Patient 4 | | | | | | | |
| Coronary branches | R_a_  (dynes s/cm^5^) | | C_a_  (cm^5^/dynes) | R_a-micro_  (dynes s/cm^5^) | C_im_  (cm^5^/dynes) | | R_v_+R_v-micro_ (dynes s/cm^5^) |
| RCA  RCA1  LAD  LAD1  LAD2  LCX | 82,316  101,541  49,228  84,976  141,835  52,989 | | 1.51e-06  1.23e-06  1.38e-06  8.03-07  4.81e-07  1.28e-06 | 133,764  165,004  79,997  138086  230,481  86,107 | 1.22e-05  9.96e-06  1.12e-05  6.50e-06  3.89e-06  1.04e-05 | | 41,158  50,770  24,614  42,488  70,917  26,494 |

RSCA: Right Subclavian Artery; RCCA: Right Common Carotid Artery; LCCA: Left Common Carotid Artery; LSCA: Left Subclavian Artery; RCA: Right Coronary Artery; LAD: Left Anterior Descending Artery; LCX: Left Circumflex Artery; R_p_: Resistance of proximal vessels; R_a_: Coronary arterial resistance; R_d_: Resistance of distal vessels; R_a-micro_: Coronary arterial microcirculation resistance; R_v_: Coronary venous resistance; R_v-micro_: Coronary venous microcirculation resistance; C: Capacitance of proximal vessels; C_a_: Coronary arterial compliance, C_im_: Intramyocardial compliance

**Comment 7: The role of TAWSS and OSI should be better discussed in terms of clinical relevance. Usually low TAWSS and high OSI refer to regions where restenosis may occur after by-pass implantation**

**Response:** As suggested, we added the following text (in bold) in the Discussion (section 4.1, page 12 & 13):

*“We showed that as the severity of native artery stenosis increases, the flow in the graft increases, resulting in marked improvement of resting and hyperemic native artery flow distal to the LIMA anastomosis (Figure 8). These computational findings were in line with clinical data from intraoperative flow measurements of native arteries and grafts post-CABG [37, 38].* ***Also, to identify areas with theoretical predisposition to atherosclerosis in the native arteries and grafts, we calculated the lumen area percentages exposed to pro-atherosclerotic low TAWSS and high OSI using the cut-off values of 1.0 Pa for low TAWSS and 0.15 for high OSI [39]. We found that with worsening stenosis, the LIMA lumen area exposed to low TAWSS was decreasing and the native lumen area (proximal to LIMA anastomoses) exposed to low TAWSS was increasing. The same pattern was observed with high OSI.****”*

**Reviewer #2:**

**In this paper the authors employ a sophisticated computational model to investigated the hemodynamics before and after CABG the main claim being that the procedure "faithfully reproduces the hemodynamic effects of bypass grafting on the native coronary artery flow".**

**I must say that I find the study interesting, though not particularly original, even if there are several fundamental questions that should be answered and points to clarify before deciding about the suitability for publication of this paper.**

We thank the Reviewer for the critique.

**Comment 1: A first relevant point is that the authors extensively use the adjective "patient specific" when referring to their simulations while they should honestly recognise that they are only loosely related to the real patients. In fact, the geometries are obtained by "manually" segmenting the lumen boundaries using an available software; this step alone introduces uncertainties and any other operator wold produce a slightly different geometry.**

**Response:** We would like to thank the Reviewer for this thoughtful comment. Our studies were based on patient-specific anatomies, which were semi-automatically segmented using a well-validated software. Our group has extensive experience with the segmentation of coronary CT angiograms, and we strongly believe that our segmentation results are highly reproducible. Indeed, the CFD parameters used were generic and not patient-specific. However, our results have been very consistent across all cases, and this gives us faith that the results would be similar if we were using patient-specific hemodynamic parameters.

We acknowledge this in the Limitations (section 4.4, page 15):

*“Sixth, there were a series of limitations related to our computational methodology: (i)* ***The values of the aortic flow rates and ventricular pressures were derived from published data corresponding to healthy individuals and not to individual study patients [27, 29].*** *(ii) To reduce the complexity of computational calculations, we used an open-loop model with the same lumped parameters in the pre- and post-CABG simulations [28], (iii) In simulations, the vascular wall was considered rigid, although we used capacitance that was distributed proportionally to the outlets to capture the global effects of flexible walls [29].”*

**Comment 2: Furthermore, the authors state that "We assumed that only 4% of the cardiac output was distributed to the coronary arteries with a 70% and 30% split in the flow between the left and the right coronary artery, respectively." These are standard data known from the literature and it is very unlikely that the real patients from which the geometries have been extracted have exactly these proportions of the flows.**

**Response:** We would like to thank the Reviewer for giving us the opportunity to clarify this point. In fact, based on the individual anatomical geometry of each patient, the distribution of coronary arteries was around 4%. The split of flow between the left and right coronary artery was determined by the individual geometry and resistance. We changed the text in the Methods (section 2.2.2a, page 6) as follows:

*“The total resting resistance of all outlets was calculated based on the ratio of mean aortic pressure to the mean flow rate.* ***We assumed that only 4% of the cardiac output was distributed to the coronary arteries to calculate the Windkessel boundary conditions at outlets.*** *The modified Murray’s law was employed to calculate the resistance of each coronary branch. [28] The total capacitance was distributed proportionally to the outlet areas."*

**Comment 3: Another choice that seems quite irrational (although it is probably suggested by computational complexity) is to consider the lumen wall rigid and then couple this non deformable geometry with Windkessel-like boundary conditions which include the compliance of the missing network of veins and arteries. If I have to think about a tract of the circulation where deformability is key is certainly the initial part of the aorta in which the blood pressure increased during systole is stored into elastic energy of the deformed walls. Finally, also the hemodynamic parameters are not specific of the two patients.**

**Response:** We thank the Reviewer for pointing out the issue of rigid wall. As alluded by the Reviewer, the main reason we considered we considered rigid wall in the CFD studies was the computational complexity. There are several publications using rigid walls for the CFD studies in the Simvascular environment, which have yielded good and consistent results *(Sankaran S, et al. Annals of Biomedical Engineering 2012;40(10):2228-42).*

We discuss the rigid wall condition in our Limitations (section 4.4, page 15):

*“Sixth, there were a series of limitations related to our computational methodology: (i) The values of the aortic flow rates and ventricular pressures were derived from published data corresponding to healthy individuals and not to individual study patients [27, 29]. (ii) To reduce the complexity of computational calculations, we used an open-loop model with the same lumped parameters in the pre- and post-CABG simulations [28], (iii)* ***In simulations, the vascular wall was considered rigid, however the capacitance was distributed proportionally to the outlets to capture the global effects of flexible walls [29].****”*

The patient-specificity of the hemodynamic parameters is discussed in our response to Comment 1.

**Comment 4: I have also important reservations about the validation of the results: the FFR values of figure 4 have little meaning if the level of confidence in those numbers is not assessed. My experience is that pressure changes sharply downstream of a stenosis and numbers can vary a lot by slightly changing the sampling position(s).**

**Response:** We thank the Reviewer for providing us the opportunity to clarify this point. According to Reviewer’s suggestion, for patient 1 and 2, we took 0.5 cm range at graft distal, and measured the FFR at 5 points with distance of 0.125 cm. And the average values were calculated. We added the measurement in the Methods (section 2.2.2b, page 6 & 7):

*“We computationally modeled the adenosine-induced hyperemia (140 mcg/kg/min) that is applied in invasive FFR measurements [31, 32]. We reduced the coronary and great vessels resistance to 22% and 95% of the resting values, respectively [33]. The FFR was computationally calculated based on the ratio of the pressure at the distal LAD to the pressure at the aortic root under simulated vasodilator-induced hyperemic conditions (Supplemental Table 4).* ***In Aim 1, the FFR was calculated distal to the LIMA anastomosis (at the same location with angiographic FFR) and averaged over a zone of 0.5 cm.*** *In Aim 2, the FFR was calculated proximally and distally to the computationally created LAD stenosis.”*

**Comment 5: The fact that the simulations are run using 2000 time steps per heartbeat, using some millions of elements, suggests that the method is numerically stabilized in some way (artificial viscosity, upwind discretizations) but there is no mention of numerical details.**

**Response:** As suggested, we summarized the solver parameters used in the CFD simulations in **Supplemental Table 3.**

**Supplemental Table 3.** Solver parameters used in the CFD simulations.

| **Parameter** | **Value** |
| --- | --- |
| Time Step Parameters | |
| Number of Timesteps | 12,000 |
| Time Step Size | 0.0005 |
| Output Control | |
| Number of Timesteps between Restarts | 100 |
| Output Surface Stress | True |
| Force Calculation Method | Velocity Based |
| Print Average Solution | True |
| Print Error Indicators | False |
| Step Construction | |
| Step Construction | 5 |
| Advanced Parameters | |
| Pressure Coupling | Implicit |
| Backflow Stabilization Coefficient | 0.2 |
| Non-linear Iteration Control | |
| Residual Control | True |
| Residual Criteria | 0.01 |
| Minimum Required Iterations | 3 |
| Linear Solver | |
| svLS Type | NS |
| Number of Krylov Vectors per Left-hand-side Formation | 100 |
| Number of Solves per Left-hand-side Formation | 1 |
| Tolerance on Momentum Equations | 0.05 |
| Tolerance on Continuity Equations | 0.4 |
| Tolerance on svLS Solver | 0.4 |
| Maximum Number of Iterations for svLS NS Solver | 1 |
| Maximum Number of Iterations for svLS Momentum Loop | 2 |
| Maximum Number of Iterations for svLS Continuity Loop | 400 |
| Discretization Control | |
| Time Integration Rule | Second Order |
| Time Integration Rho Infinity | 0.5 |
| Flow Advection Form | Convective |
| Quadrature Rule on Interior | 2 |
| Quatrature Rule on Boundary | 3 |

**Comment 6: To conclude, the study might be interesting but, as is, there are too many unclear points, unjustified statements and bold claims to recommend publication.**

**Response:** We hope the Reviewer is now satisfied with our detailed responses to the comments raised.
